# Unchanged Severity of Influenza A(H1N1)pdm09 Infection in Children during First Postpandemic Season

**DOI:** 10.3201/eid1811.120719

**Published:** 2012-11

**Authors:** Mathias Altmann, Lena Fiebig, Silke Buda, Rüdiger von Kries, Manuel Dehnert, Walter Haas

**Affiliations:** Robert Koch Institute, Berlin, Germany (M. Altmann, L. Fiebig, S. Buda, M. Dehnert, W. Haas);; and Ludwig-Maximilians-Universität, München, Germany (R. von Kries)

**Keywords:** influenza A(H1N1)pdm09 virus, influenza, children, critically ill, postpandemic, hospital-acquired infection, nosocomial transmission, viruses

## Abstract

We conducted a nationwide hospital-based prospective study in Germany of influenza A(H1N1)pdm09 cases among children <15 years of age admitted to pediatric intensive care units and related deaths during the 2009–10 pandemic and the 2010–11 postpandemic influenza seasons. We identified 156 eligible patients: 112 in 2009–10 and 44 in 2010–11. Although a shift to younger patients occurred in 2010–11 (median age 3.2 vs. 5.3 years), infants <1 year of age remained the most affected. Underlying immunosuppression was a risk factor for hospital-acquired infections (p = 0.013), which accounted for 14% of cases. Myocarditis was predictive of death (p = 0.006). Of the 156 case-patients, 17% died; the difference between seasons was not significant (p = 0.473). Our findings stress the challenge of preventing severe postpandemic influenza infection in children and the need to prevent nosocomial transmission of influenza virus, especially in immunosuppressed children.

## Abstract 2

Improvement is needed in preventing severe disease and nosocomial transmission in children beyond pandemic situations.

In Germany during the influenza A(H1N1)pdm09 pandemic, there were ≈1,070,000 influenza-related medical consultations and ≈1,800 hospitalizations for children 0–14 years of age during October 12, 2009–January 15, 2010, as determined using data provided by the German syndromic surveillance system for acute respiratory infections (*1*). Moreover, 29 laboratory-confirmed A(H1N1)pdm09 infection–related deaths in children were notified through the mandatory German surveillance system for infectious diseases (*2*). The highest number of notified hospitalizations and deaths were among children 10–14 years of age (*3*,*4*). In a nationwide hospital-based observational study investigating severely ill children who had been admitted to pediatric intensive care units (PICUs) or had died with laboratory-confirmed A(H1N1)pdm09, we reported a high proportion (75%) of case-patients with underlying risk factors, of which neurodevelopmental disorders were most prevalent (*5*). In addition, we found that in 10% of the cases, children had acquired their infection while hospitalized and that few had been vaccinated, revealing a need for improving preventive measures to reduce severe disease and adverse outcomes (*5*).

On August 10, 2010, the general director of the World Health Organization declared the world was no longer in phase 6 of influenza pandemic alert; we were moving into the postpandemic phase (*6*). Experience from past pandemics suggested that the pandemic virus would gradually take on the behavior of a seasonal influenza virus and circulate for several years. However, in view of the potential for transformation of the virus into a more virulent form (*7*), as suggested by higher rates of mortality during second pandemic waves in Copenhagen (1918), the United States (1957), and Eurasia (1968–1970) (*8*,*9*), the World Health Organization acknowledged the unpredictability of pandemic viruses; recommended continued vigilance; and issued advice on surveillance, vaccination, and prompt clinical management of cases during the postpandemic phase (*6*).

Little is known about the severity of A(H1N1)pdm09 in children during the first postpandemic season (*10*). To obtain information on critically ill A(H1N1)pdm09-infected children and to compare risk factors and disease course, outcome, and severity for patients during the pandemic and first postpandemic season, we prospectively and continuously performed a nationwide study in Germany during August 3, 2009–July 29, 2011.

## Methods

### Study Design

We conducted a nationwide prospective observational study in Germany by using the German Survey Center for Rare Pediatric Diseases (ESPED; an established children’s hospitals network comprising all 375 pediatric hospitals in Germany) to identify children <15 years of age admitted to PICUs with confirmed A(H1N1)pdm09 infection and related deaths. Cases and related deaths during August 3, 2009–July 29, 2011 were reported by using a standardized form. In pandemic season 2009–10 (August 3, 2009–August 9, 2010), the case definition included only A(H1N1)pdm09 infection; in postpandemic season 2010–11 (August 10, 2010–July 29, 2011), the case definition included all influenza virus infections.

### Data Collection

**Figure 1:**
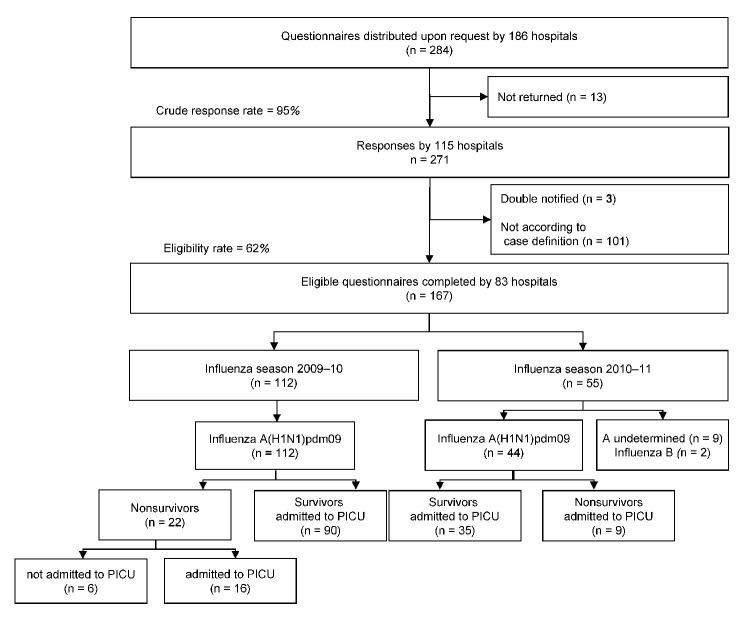
Overview of study participation and participant groups among severe pediatric cases with A(H1N1)pdm09, Germany, 2009–2011. PCIU, pediatric intensive care unit.

On notification by treating physicians of patients with A(H1N1)pdm09 infection, the ESPED study center distributed and subsequently collected a structured questionnaire, which had been adapted by the authors from an earlier study on seasonal influenza (*11*). Of 284 distributed questionnaires requested by 186 hospitals, 95% (271/284) were returned to the study center (Figure 1). After excluding 3 questionnaires that had been notified twice and 101 questionnaires for patients who did not meet the case definition, 62% (167/271) of the questionnaires from 83 hospitals remained. Reasons for not meeting the case definition included patient age >15 years and patient not admitted to PICU. In accordance with the case definition, only cases of A(H1N1)pdm09 infection were reported during the pandemic season (2009–10), and 9 cases of influenza A (not further subtyped) and 2 cases of influenza B infection were reported in the postpandemic season (2010–11). Therefore, further analyses were restricted to A(H1N1)pdm09 cases.

The structured questionnaire covered anonymous patient information and information regarding the hospital stay, clinical signs and symptoms, clinical and laboratory findings, specific treatments, status of influenza vaccination, disease complications, and underlying chronic medical conditions (chronic respiratory diseases; cardiac diseases; immunodeficiency; and neurodevelopmental disorders, including developmental delay, cerebral palsy, epilepsy, and other cognitive disorders). Answer categories were predetermined, but free space was designated for respondents to provide information about other diagnoses and coexisting illnesses/medical conditions. Hospital-acquired infection was defined by a date of symptom onset being >2 days after the date of hospital admission; 2 days corresponds with the median incubation time for A(H1N1)pdm09 according to Cao et al. (*12*). Data were double entered by using EpiData 3.1 software (www.epidata.dk/) in an electronic database.

### Data Analysis

Reported values are those for children with available information. Descriptive statistics comprised the calculation of median and interquartile ranges (IQRs) for continuous variables and absolute numbers and proportions (together with 95% binomial exact CIs, when appropriate) for categorical variables. Comparative analyses were based on the Wilcoxon rank-sum test for continuous variables and Fisher exact test for categorical variables. Odds ratios (ORs) and 95% CIs were calculated. Multivariable analysis was performed by using a logistic regression with a stepwise approach to compare cases of hospital-acquired infection with cases of community-acquired infection and survivors with nonsurvivors in PICUs. In doing so, risk factors with a p value <0.2 were considered for multivariable analysis, with the exception of age, sex, and season, which were included in all models. Reported p values are 2-sided, and p<0.05 was considered significant. Statistical analyses were performed by using Stata 11.0 (StataCorp LP, College Station, TX, USA).

### Data Protection and Ethics Clearance

Adherence to national data protection laws was approved by the Federal Commissioner for Data Protection and Freedom of Information of Germany. Ethical approval was granted by the Ethics Committee, Charité-Universitätsmedizin, Berlin, Germany.

## Results

### Comparison of 2009–10 and 2010–11 Seasons

**Figure 2:**
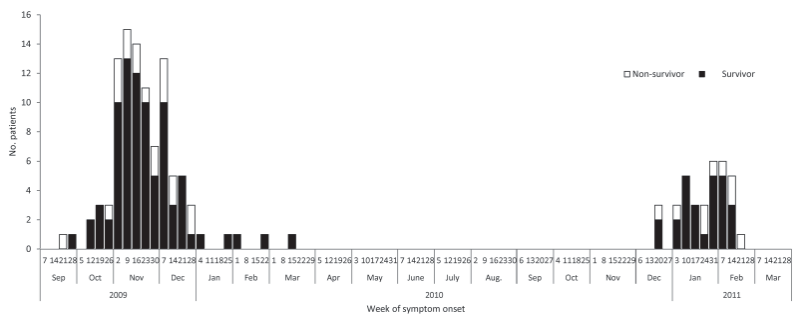
Distribution of 136 critically ill children with confirmed A(H1N1)pdm09, by date of disease onset, September 21, 2009–February 22, 2011, Germany. Only cases with available date of symptom onset are represented.

We identified 156 critically ill children with confirmed A(H1N1)pdm09 infection: 112 in 2009–10 and 44 in 2010–11 (Figure 1). Dates of symptom onset ranged from September 21, 2009, to March 20, 2010, for 2009–10 and from December 20, 2010, to February 22, 2011, for 2010–11 (Figure 2). Cases were reported from 15 of the 16 federal states in Germany during 2009–10 and from 10 during 2010–11.

**Figure 3:**
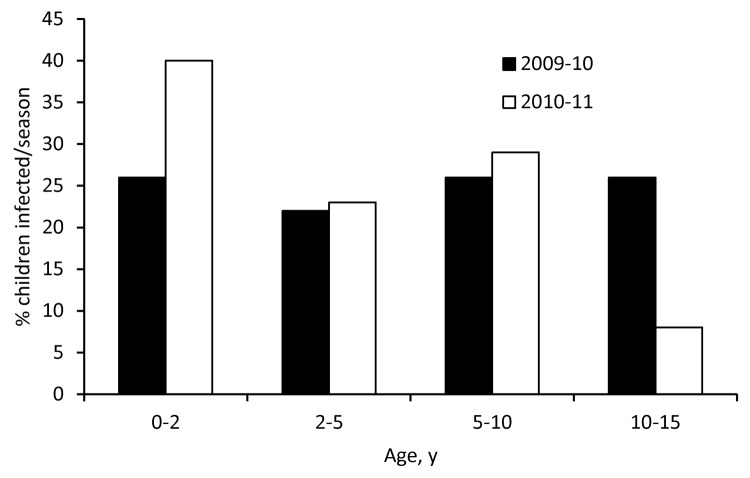
Proportion of critically ill children with A(H1N1)pdm09 by age group and season, Germany.

**Figure 4:**
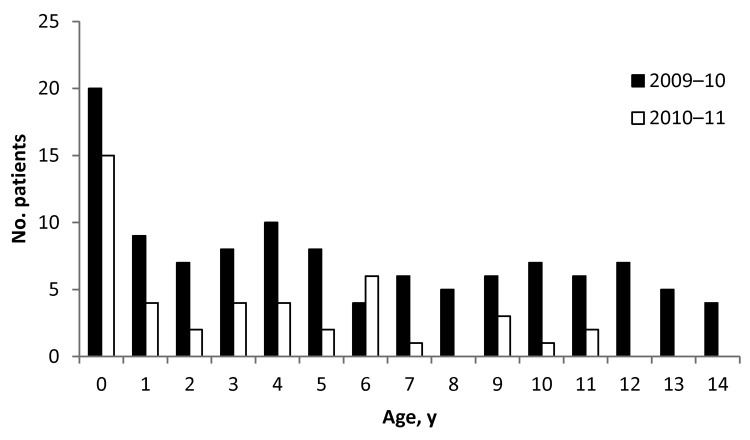
Age distribution of the 156 critically ill children with confirmed A(H1N1)pdm09, by season, Germany.

The proportion of boys among case-patients was higher in 2009–10 than 2010–11 (59% vs. 37%, p = 0.02) (Table 1). The median age of case-patients was 5.3 and 3.2 years in 2009–10 and 2010–11, respectively, and differed statistically (p = 0.007) between the 2 seasons. The age distribution in 2010–11 compared with that in 2009–10 was characterized by a markedly higher proportion of children < 2 years of age and a lower proportion of children 10–14 years of age (Figure 3). In both seasons, infants <1 year of age represented the age group with the highest number of cases (Figure 4).

**Table 1:** Comparison of severe cases of influenza A(H1N1)pdm09 virus infection in children during the pandemic and the first postpandemic seasons, Germany, 2009–2011*

Variable	No. patients/no. total (%)	Influenza season		p value
		2009–10	2010–11	
Male sex	81/154 (53)	65/111 (59)	16/43 (37)	0.020
Median age, y (IQR)	4.2 (1.2–9.2)	5.3 (1.7–10.1)	3.2 (0.5–6.5)	0.007
Hospital-acquired infection	19/136 (14)	11/101 (11)	8/35 (23)	0.093
Clinical diagnosis				
Pneumonia	108/156 (69)	79/112 (71)	29/44 (66)	0.569
Secondary pneumonia	30/156 (19)	22/112 (20)	8/44 (18)	1.000
Encephalopathy	11/156 (7)	7/112 (6)	4/44 (9)	0.506
ARDS	43/156 (28)	29/112 (26)	14/44 (32)	0.551
Sepsis	18/156 (12)	9/112 (8)	9/44 (21)	0.048
Myocarditis	8/156 (5)	4/112 (4)	4/44 (9)	0.223
Febrile seizure	7/156 (5)	3/112 (3)	4/44 (9)	0.099
Underlying chronic medical conditions				
Any	114/146 (78)	82/107 (77)	32/39 (82)	0.652
Neurodevelopmental disorders	84/151 (56)	61/110 (56)	23/41 (56)	1.000
Respiratory disease	44/141 (31)	35/104 (34)	9/37 (24)	0.409
Immunodeficiency	17/137 (12)	15/97 (16)	2/40 (5)	0.152
Cardiac disease	20/143 (14)	12/102 (12)	8/41 (20)	0.286
Treatment				
Oseltamivir	90/145 (62)	65/105 (62)	25/40 (63)	1.000
Catecholamine	52/138 (38)	35/101 (35)	17/37 (46)	0.240
Mechanical ventilation	98/145 (68)	68/107 (64)	30/38 (79)	0.107
Vaccination†	5/88 (6)	5/67 (8)	0/21 (0)	0.332
Outcome				
All death	31/156 (20)	22/112 (20)	9/44 (21)	1.000
Death in PICU	25/150 (17)	16/106 (15)	9/44 (21)	0.473

*Values are no. positive/no. with available information (%), except as indicated. Pandemic season, 2009–10; postpandemic season, 2010–11; IQR, interquartile range; ARDS, acute respiratory distress syndrome; PICU, pediatric intensive care unit. †Influenza A(H1N1)pdm09 vaccination of patients >6 mo of age.

Of the 146 children with available information, 114 (78%) had >1 chronic underlying medical condition; the difference between seasons for these conditions was not statistically significant (Table 1). In both seasons, neurodevelopmental disorders were the most prevalent underlying medical condition. Of the 156 critically ill case-patients, 130 were >6 months age and thus eligible for vaccination against A(H1N1)pdm09 virus; however, for children with available information on vaccination status, only 5 (7%) of 67 vaccine-eligible case-patients had been vaccinated in the 2009–10 season, and none had been vaccinated in the 2010–11 season. Of the 69 total children in both seasons with underlying chronic medical conditions, 64 (93%) had not been vaccinated against A(H1N1)pdm09 virus.

More cases of sepsis were reported during the postpandemic season than during the pandemic season (21% vs. 8%; p = 0.048) (Table 1). Treatment with oseltamivir was used equally (in ≈62% of children) during both seasons. The time to oseltamivir administration after symptom onset (median 4 days) was similar throughout both seasons. The use of catecholamine and mechanical ventilation was more frequent in 2010–11 than in 2009–10, but the difference was not statistically different.

### Hospital-acquired Infections

Hospital-acquired infections accounted for 11% (11 of 101) of the cases in 2009–10 and for 23% (8/35) in 2010–11 (p = 0.0931) (Table 1). Of the total study cohort, 14% (19/136) of the patients (9 in a general ward and 10 in a PICU) most likely had hospital-acquired infection. For these case-patients, the median time from hospital admission to symptom onset was 29 days (IQR 12–73 days). The median age for patients with hospital-acquired infection was 1.1 years, and 56% (10/18) were boys (difference not statistically significant between seasons).

The overall case-fatality ratios were 26% (5/19) among patients with hospital-acquired infection and 20% (23/117) among those with community-acquired infection (p = 0.543). Compared with patients with community-acquired infection, those with hospital-acquired infection had more complications, including acute respiratory distress syndrome (ARDS) (OR 2.7, p = 0.054) and sepsis (OR 3.1, p = 0.064), but the differences were not statistically significant (Table 2). In the multivariable model, immunodeficiency (OR 5.9, 95% CI 1.5–23.9; p = 0.013) and mechanical ventilation (OR 8.9, 95% CI 1.1–74.7; p = 0.043) were significantly associated with hospital-acquired infection after adjusting for age, sex, and season.

**Table 2:** Comparison of hospital- and community-acquired cases of severe influenza A(H1N1)pdm09 infection in children during the pandemic and first postpandemic influenza seasons, Germany, 2009–2011*

Variable	No. patients/no. total (%)	Hospital-acquired cases	Community-acquired cases‡	Univariable analysis			Multivariable analysis	
				OR (95% CI)	p value		OR (95% CI)	p value
Male sex	72/134 (54)	10/18 (56)	62/116 (54)	1.1 (0.4–3.4)	1.000		1.4 (0.4–4.5)	0.622
Median age, years (IQR)	4.3 (1.0–9.2)	1.1 (0.3–10.1)	4.8 (1.6–11.9)	NA	0.168		1.0 (0.8–1.1)	0.583
2010–11 season	35/136 (26)	8/19 (42)	27/117 (23)	2.4 (0.8–7.4)	0.093		1.5 (0.4–5.7)	0.517
Clinical diagnosis								
Pneumonia	96/136 (71)	15/19 (79)	81/117 (69)	1.7 (0.5–7.4)	0.588		NA	NA
Secondary pneumonia	27/136 (20)	4/19 (21)	23/117 (20)	1.1 (0.2–3.8)	1.000		NA	NA
Encephalopathy	9/136 (7)	0/19 (0)	9/117 (8)	0.0 (0.0–2.5)	0.360		NA	NA
ARDS	38/136 (28)	9/19 (47)	29/117 (25)	2.7 (0.9–8.2)	0.054		NA	NA
Sepsis	17/136 (13)	5/19 (26)	12/117 (10)	3.1 (0.7–11.4)	0.064		NA	NA
Myocarditis	8/136 (6)	0/19 (0)	8/117 (7)	0.0 (0.0–2.9)	0.600		NA	NA
Febrile seizure	6/136 (4)	0/19 (0)	6/117 (5)	0.0 (0.0–3.9)	0.595		NA	NA
Underlying chronic medical conditions								
Any	101/129 (78)	19/19 (100)	82/110 (75)	NA (1.7–NA)	0.013		NA	NA
Neurodevelopmental disorders	75/133 (56)	13/19 (68)	62/114 (54)	1.8 (0.6–6.2)	0.321		NA	NA
Respiratory disease	17/126 (14)	3/18 (17)	14/108 (13)	1.3 (0.2–5.7)	0.710		NA	NA
Immunodeficiency	15/121 (12)	5/17 (29)	10/104 (10)	3.9 (0.9–15.2)	0.037		5.9 (1.5–23.9)	0.013
Cardiac disease	17/126 (14)	3/18 (17)	14/108 (13)	1.3 (0.2–5.7)	0.710		NA	NA
Treatment								
Oseltamivir	80/128 (63)	13/18 (72)	67/110 (61)	1.7 (0.5–6.4)	0.4378		NA	NA
Catecholamine	47/124 (38)	11/16 (69)	36/108 (33)	4.4 (1.3–17.2)	0.011		NA	NA
Mechanical ventilation	85/129 (66)	17/18 (94)	68/111 (61)	10.8 (1.6–459.4)	0.006		8.9 (1.1–74.7)	0.043
Vaccination†	5/80 (6)	0/10 (0)	5/70 (7)	0.0 (0.0–5.5)	1.000		NA	NA
Outcome								
All deaths	28/136 (21)	5/19 (26)	23/117 (20)	1.5 (0.4–4.9)	0.543		NA	NA
Death in PICU	22/130 (17)	4/18 (22)	18/112 (16)	1.5 (0.3–5.5)	0.507		NA	NA

*Values are no. positive/no. with available information (%), except as indicated. Pandemic season, 2009–10; postpandemic season, 2010–11; OR, odds ratio; IQR, interquartile range; NA, not applicable; ARDS, acute respiratory distress syndrome; PICU, pediatric intensive care unit. †Influenza A(H1N1)pdm09 vaccination of patients >6 mo of age..

### Case Fatalities Ratios

The case fatality ratio in PICUs did not differ between seasons: 15% (16/106) and 21% (9/44) of PICU case-patients died in 2009–10 and 2010–11, respectively (p = 0.473) (Table 1). For the 2 seasons, 25 of 150 PICU case-patients died, corresponding to a case-fatality ratio of 17% (95% CI 11%–24%). On hospital discharge, 26% (27/104) of the survivors were reported to have possible sequelae or worsening of a pre-existing medical condition.

No statistical differences were found between survivors and nonsurvivors in underlying chronic medical conditions and vaccination status. ARDS (OR 3.2, 95% CI 1.1–9.2, p = 0.029), myocarditis (OR 30.9, 95% CI 2.6–360.7,; p = 0.006), and mechanical ventilation (OR 18.3; 95% CI 1.3–251.6, p = 0.030) were independently associated with a fatal outcome in the multivariable model after adjusting for age, sex, and season (Table 3).

**Table 3:** Comparison of severe cases of influenza A(H1N1)pdm09 infection among children in PICUs during the pandemic and first postpandemic influenza seasons, Germany, 2009–2011*

Variable	No. patients/no. total (%)	Nonsurvivors	Survivors‡	Univariable analysis			Multivariable analysis	
				OR (95% CI)	p value		OR (95% CI)	p value
Male sex	81/148 (55)	15/25 (60)	66/123 (54)	1.3 (0.5–3.5)	0.661		2.5 (0.8–7.4)	0.098
Median age, years (IQR)	4.2 (1.0–8-6)	5.7 (1.6–9.8)	4.1 (1–7.8)	NA	0.091		1.1 (1.0–1.2)	0.232
2010–11 season	44/150 (29)	9/25 (36)	35/125 (28)	1.4 (0.5–3.9)	0.4728		1.6 (0.5–5.1)	0.435
Hospital-acquired infection	18/130 (14)	4/22 (18)	14/108 (13)	1.5 (0.3–5.5)	0.507		NA	NA
Clinical diagnosis								
Pneumonia	103/150 (69)	13/25 (52)	90/125 (72)	0.4 (0.2–1.1)	0.060		NA	NA
Secondary pneumonia	30/150 (20)	8/25 (32)	22/125 (18)	2.2 (0.7–6.2)	0.108		NA	NA
Encephalopathy	11/150 (7)	1/25 (4)	10/125 (8)	0.5 (0.01–3.7)	0.692		NA	NA
ARDS	43/150 (29)	15/25 (60)	28/125 (22)	5.2 (1.9–14.3)	<0.001		3.2 (1.1–9.2)	0.029
Sepsis	17/150 (11)	6/25 (24)	11/125 (9)	3.3 (0.9–11.0)	0.040		NA	NA
Myocarditis	8/150 (5)	4/25 (16)	4/125 (3)	5.8 (1.0–32.9)	0.027		30.9 (2.6–360.7)	0.006
Febrile seizure	7/150 (5)	0/25 (0)	7/125 (6)	0.0 (0.01–2.7)	0.601		NA	NA
Underlying chronic medical condition								
Any	108/140 (77)	19/23 (83)	89/117 (76)	1.5 (0.4–6.5)	0.596		NA	NA
Neurodevelopmental disorder	78/145 (54)	16/24 (67)	62/121 (51)	1.9 (0.7–5.5)	0.186		NA	NA
Respiratory disease	42/135 (31)	8/22 (36)	34/113 (30)	1.3 (0.4–3.8)	0.617		NA	NA
Immunodeficiency	16/131 (12)	1/21 (5)	15/110 (14)	0.3 (0.0–2.3)	0.467		NA	NA
Cardiac disease	20/137 (15)	4/23 (17)	16/114 (14)	1.3 (0.3–4.6)	0.746		NA	NA
Treatment								
Oseltamivir	87/139 (63)	15/23 (65)	72/116 (62)	1.1 (0.4–3.4)	0.8185		NA	NA
Catecholamine	52/133 (39)	16/23 (70)	36/110 (33)	4.7 (1.6–14.6)	0.002		NA	NA
Mechanical ventilation	98/140 (70)	23/24 (96)	75/116 (65)	12.6 (1.9–530.3)	0.001		18.3 (1.3–251.6)	0.030
Vaccination†	5/82 (6)	0/14 (0)	5/68 (7)	0.0 (0.0–3.7)	0.582		NA	NA

*Values are no. positive/no. with available information (%), except as indicated. PICUs, pediatric intensive care units; pandemic season, 2009–10; postpandemic season, 2010–11; OR, odds ratio; IQR, interquartile range; NA, not applicable; ARDS, acute respiratory distress syndrome. †Influenza A(H1N1)pdm09 vaccination of patients >6 mo of age.

Compared with survivors, nonsurvivors more frequently required mechanical ventilation (p = 0.001) and treatment with catecholamine (p = 0.002); no differences were found in oseltamivir administration (65% vs. 62%, p = 0.8185). Time from symptom onset to oseltamivir uptake did not differ between survivors (median 4 days, IQR 1–6 days) and nonsurvivors (median 4 days, IQR 2–8 days).

## Discussion

During the first postpandemic season, fewer cases of A(H1N1) pdm09 infection were reported, but the severity and outcome of cases did not differ between the pandemic and postpandemic seasons. We further analyzed data from the 2 seasons as 2 outbreak waves of 1 virus and identified a high number of hospital-acquired infections and ARDS and myocarditis as 2 predictors for a fatal outcome.

Compared with the 2009–10 pandemic season, the 2010–11 postpandemic season started later in the winter and had less than half the number of cases. High disease awareness during the pandemic season may have enhanced testing and reporting during 2009–10; thus the reduced case number for 2010–11 should be interpreted with caution. However in the United States, where reporting of influenza-related deaths in children is mandatory, a similar decline in the number of fatal A(H1N1)pdm09-associated cases was noted between the 2009–10 and 2010–11 influenza seasons (282 and 71 deaths among children, respectively) (*13*). Before the 2009–10 pandemic and similar to the postpandemic season, an average of 82 (range 46–153) children in the United States died each year from seasonal influenza–related illnesses (*14*). However, in the postpandemic 2010–11 season, different proportions of all circulating influenza subtypes might have led to different numbers of persons exposed to A(H1N1)pdm09, which makes comparisons between seasons and across countries difficult.

For the 2010–11 season, we assumed a more limited number of susceptible persons because exposure to influenza virus during the pandemic might have provided immunologic protection (*15*–*17*). This hypothesis is supported by our results showing a shift toward infection in younger age groups in 2010–11. A similar finding was reported in a prospective study of children with A(H1N1)pdm09 infection in a Spanish hospital (median age 7.0 and 0.8 years in 2009–10 and 2010–11, respectively) (*10*). During both seasons, children <1 year of age were more affected than those in other age groups, and the numbers infected in the 2 seasons were similar; therefore, influenza infection in this immunologically naive age group might always reflect a pandemic-like situation. It remains unexplained why more boys in 2009–10 (59%, 65/111) than in 2010–11 (37%, 16/43) had serious A(H1N1)pdm09 infection, although it has been suggested that the difference in age distribution between the 2 seasons could have influenced the sex distribution (*18*).

Our results show that case-fatality ratios for the 2 seasons were similar. In Greece (*19*) and New-Zealand (*20*), according to the respective national surveillance systems in intensive care unit settings, case-fatality ratios among all age groups were also similar for the 2 seasons. This result is reassuring, in view of concerns of a possible transformation of the strain into a more severe form (*7*), and is in agreement with the antigenic and genetic homogeneity of the virus since its emergence (*21*).

In both seasons, we identified a large number of probable hospital-acquired A(H1N1)pdm09 infections. Immunodeficiency, most often reported as acute lymphoblastic leukemia, was associated with hospital-acquired infection, and this underlying chronic medical condition, has also been identified as a risk factor for community-acquired A(H1N1)pdm09 (*22*,*23*). Findings from a retrospective hospital-based study investigating the prevalence of respiratory virus infections among children with cancer or HIV infection reported that 40% of the respiratory infections were acquired during the hospital stay, and influenza A virus was the second most prevalent respiratory infection (*24*). In our study, patients with hospital-acquired infection had more complications, including ARDS and sepsis, than patients with community-acquired infections. However, a significant association between hospital-acquired infection and death was not found, possibly because of the small number of cases, as found by Spaeder et al. (*25*) in a retrospective cohort study in PICU setting. In this study, hospital acquisition of viral respiratory infection was shown to be associated with an increased risk for death, even after adjusting for chronic medical conditions that predispose to an increased risk for complications from viral illness. Our findings emphasize the need for isolation of and preventive measures for children with immunodeficiency, as reported (*26*). Preventive measures should include the vaccination of health care workers. Indeed, a survey in Germany showed that only 35% (n = 3,900) of the health care workers in a university hospital setting were vaccinated during the 2010–11 influenza season (*27*). Studies in earlier seasons showed even lower influenza vaccination rates among health care workers (*28*).

We identified 25 A(H1N1)pdm09-associated deaths among children admitted to PICUs during the pandemic and postpandemic seasons. ARDS was the most prevalent complication among case-patients who died (60% of cases) and was highly associated with death. Myocarditis was also highly associated with death in children; this finding supports those among adults (*29*,*30*) and other findings among A(H1N1)pdm09-infected children (*31*).

During both seasons, 62% of the children received oseltamivir treatment. This proportion is lower than described in other studies in PICU settings, e.g., 81% in an inception-cohort study in Australia and New Zealand (*32*), 88% in a US cohort (*31*), 96% in a retrospective observational multicenter study in Turkey (*33*), and 100% of children in a retrospective Dutch cohort (*34*). Observational and random clinical trials have shown the potential of oseltamivir to reduce the length of hospitalization when started <24 hours of illness onset (*35*,*36*). The Infectious Diseases Society of America recommends that any person with confirmed or suspected influenza who requires hospitalization receive influenza antiviral therapy, even if the patient enters care >48 hours after illness onset (*37*). The German Society for Pediatric Infectious Diseases recommends that immunocompetent children without underlying chronic medical conditions should not receive influenza antiviral therapy >48 hours after onset of influenza symptoms (*38*). Most A(H1N1)pdm09 virus isolates tested worldwide remain sensitive to oseltamivir; thus, strategies to optimize the use of oseltamivir should be considered, and additional evidence should be collected with respect to reduction of nosocomial spread of A(H1N1)pdm09 virus and to potential benefits from late treatment in severely ill children.

We showed that 93% of the children with underlying chronic medical conditions who were eligible for vaccination had not been vaccinated. This finding highlights a need to improve vaccine coverage among this population, for which influenza vaccination is recommended in Germany (*39*). Children who did not survive received more intensive treatment (mechanical ventilation and catecholamine) than those who survived, and nearly all influenza A viruses tested continue to be antigenically similar to those found in the current trivalent vaccine (*40*); thus, enhanced prevention in children through vaccination, especially among those with underlying chronic medical conditions, remains a high priority.

Our study is subject to several limitations. Factors such as physicians’ awareness, diagnostic testing, and reporting behavior, which may have had different influences in the 2 seasons, were not assessed. Only children hospitalized in pediatric hospitals were included in the study; however, it can be assumed that critically ill children hospitalized in general hospitals were transferred to pediatric hospitals covered by the ESPED network. In addition, our knowledge of the clinical features of patients was based only on information provided in the structured questionnaires. Ascertainment of underlying chronic medical conditions was not standardized and, thus, may have differed among treating physicians.

## Conclusions

During the first postpandemic A(H1N1)pdm09 season, the situation for children with severe A(H1N1)pdm09 disease did not differ from that for children with severe disease during the pandemic. Signs of pulmonary failure or suspected myocarditis in such children should alert health care providers to immediately initiate maximum care, and prevention of nosocomial transmission of influenza virus should be reinforced, especially in immunosuppressed children. The unchanged severity of influenza A(H1N1)pdm09 virus infections in the first postpandemic season (2010–11) and the constant high proportion of possibly hospital-acquired infections stress the challenge of preventing severe cases in children beyond the pandemic situation.
